# NRIP3 upregulation confers resistance to chemoradiotherapy in ESCC via RTF2 removal by accelerating ubiquitination and degradation of RTF2

**DOI:** 10.1038/s41389-020-00260-4

**Published:** 2020-08-24

**Authors:** Daqin Suo, Ling Wang, Tingting Zeng, Hui Zhang, Lei Li, Jinyun Liu, Jingping Yun, Xin-Yuan Guan, Yan Li

**Affiliations:** 1 State Key Laboratory of Oncology in South China, Collaborative Innovation Center for Cancer Medicine, Sun Yat-sen University Cancer Center, Guangzhou, China; 2 Metabolic Innovation Center, Zhongshan School of Medicine, Sun Yat-sen University, Guangzhou, Guangdong China; 3 Department of Clinical Oncology, University of Hong Kong, Hong Kong, China; 4 Guangdong Esophageal Cancer Institute, Guangdong, China

**Keywords:** Cancer genetics, Gastrointestinal cancer

## Abstract

Esophageal squamous cell carcinoma (ESCC) is a common malignant cancer worldwide. Despite recent improvements in surgical techniques and adjuvant therapies, the prognosis of patients with advanced ESCC remains poor. Resistance to chemoradiotherapy (CRT) remains a major cause of treatment failure for advanced ESCC patients. Here, we report that NRIP3 (nuclear receptor interacting protein 3) promotes ESCC tumor cell growth and resistance to CRT in ESCC cells by increasing and binding to DDI1 (DNA-damage inducible 1 homolog 1) and RTF2 (homologous to *Schizosaccharomyces* *pombe* Rtf2), and accelerating the removal of RTF2, which is a key determinant for the ability of cells to manage replication stress. In addition, we found that NRIP3 could increase DDI1 expression via PPARα. The NRIP3-PPARα-DDI1-RTF2 axis represents a protective molecular pathway in ESCC cells that mediates resistance to replication stress signals induced by chemoradiotherapy. In addition, elevated NRIP3 is associated with the poor clinical outcome of ESCC patients receiving radiotherapy and/or cisplatin-based chemotherapy. Our study therefore reveals that NRIP3 is a prognostic factor in ESCC and could have some predictive value to select patients who benefit from CRT treatment. A common mechanism that protects ESCC tumor cells from DNA damage induced by CRT is also revealed in this study.

## Introduction

Esophageal squamous cell carcinoma (ESCC) is a common malignant cancer worldwide. Despite recent improvements in surgical techniques and adjuvant therapies, the prognosis of patients with advanced ESCC remains poor^1^. Recently, the results of the ChemoRadiotherapy (CRT) for Esophageal cancer followed by Surgery Study comparing neoadjuvant chemoradiotherapy (nCRT) plus surgery versus surgery alone in patients with ESCC showed a significant increase in overall survival in favor of the nCRT plus surgery group^2,3^. However, locoregional recurrence remains a major cause of treatment failure and develops in 40–60% of patients^4^. Given the importance of CRT resistance in ESCC recurrence, a better understanding of the mechanism of CRT resistance will provide critical information for predicting the prognosis and selecting the appropriate treatment regimen for patients with ESCC.

Nuclear receptors are ligand-activated transcription factors that directly regulate genes and are involved in many physiological processes, including growth, development, homeostasis, differentiation, and metabolism^5^. Currently, nuclear receptors represent one of the largest transcription factor families, and nuclear receptor signaling dysregulation contributes to various human diseases, such as cancer^6^. NRIP1 (nuclear receptor interacting protein 1) is a transcriptional coregulator that interacts with many nuclear receptors, including estrogen receptor, peroxisome proliferator-activated receptors (PPARs), etc.^7^. It acts as either a corepressor or coactivator to regulate numerous biological processes^8^. Unlike many studies indicating that NRIP1 and NRIP2 are associated with the progression and development of cancers^9–11^, NRIP3 (nuclear receptor interacting protein 3) has not been investigated much. A transforming *MLL-NRIP3* fusion gene was identified in acute leukemia, and retrovirus-mediated ectopic expression of *MLL-NRIP3* in mouse hematopoietic cells was able to induce myeloid leukemia^12,13^. NRIP3 was also identified as one of the key molecular targets significantly correlated with the prognosis of breast cancer by bioinformatics analysis^14^. In the bioinformatics analysis of genes in gastric mucosa associated with obesity and obesity-related diabetes, NRIP3 was identified as one of the differentially expressed genes that played crucial roles in metabolic, T-cell, and G-protein coupled receptor biological processes and Age–Rage signaling^15^.

**Fig. 1:**
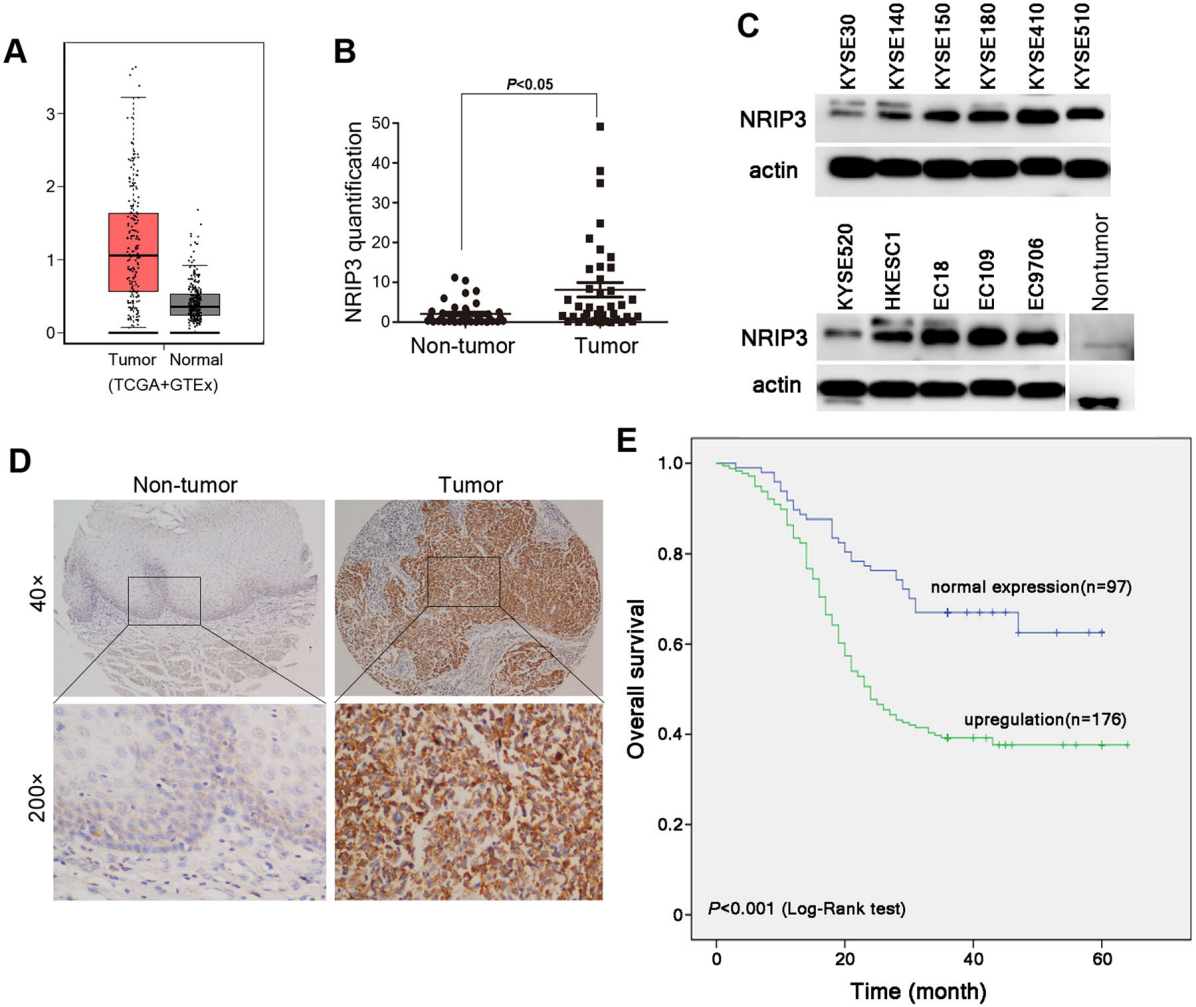
NRIP3 is upregulated in ESCC tumor tissues. **a** The NRIP3 expression profile was compared between ESCC tumors and normal tissues (TCGA + GTEx datasets). **b** Relative quantification of NRIP3 was determined in 40 pairs of ESCC tumor tissues and nontumor tissues. The results were normalized to the β-actin mRNA level. **c** The protein level of NRIP3 was determined in ESCC cell lines and a nontumor tissue pool (ten cases of nontumor tissues). **d** Representative IHC images of NRIP3 in a pair of ESCC tumor tissue and nontumor tissue. **e** Kaplan–Meier plots of overall survival of patients stratified by expression of NRIP3 (log-rank test).

Despite the identification of NRIP3 by bioinformatics analysis and the fact that NRIP3 is highly expressed in many tumors according to the public database (Fig. S1A), relatively little is known about the role and function of NRIP3 in cancer. Based on our previous cDNA microarray data of ESCC^16^, NRIP3 was upregulated in ESCC tumor tissues compared with nontumor tissues. The gene expression profiling from big database (TCGA) also indicates its upregulation in ESCC tumor tissues (Fig. 1a). In this study we found an important role of NRIP3 in progression and CRT resistance of ESCC tumor cells. We also identified a critical role of NRIP3 in increasing the proteasomal shuttle protein DDI1 and promoting RTF2 (replication termination factor 2 (RTF2), homologous to *Schizosaccharomyces pombe* Rtf2) removal for replication fork restart in the DNA damage reaction. As literatures suggest, RTF2 mediates replication termination by inhibiting replication start and RTF2 removal is important for replication fork restart and progression^17,18^. Excess RTF2 is detrimental to replication fork progression during replication stress^18^. The axis that NRIP3 upregulates DDI1 via PPARα and then promotes RTF2 removal represents a protective molecular pathway in cancer cells that mediates their resistance to replication stress signals induced by CRT.

## Results

### NRIP3 upregulation is correlated with poorer prognosis of patients with ESCC

The gene expression profile across the ESCC tumor samples from TCGA database and normal samples from GTEx indicates that NRIP3 is upregulated in ESCC tumor samples (Figs. 1a and S1A). qRT-PCR was performed to evaluate the mRNA level of NRIP3 in 40 pairs of ESCC tumor and nontumor tissues. NRIP3 was upregulated in 57.5% (23/40) of ESCC tumor tissues compared with nontumor tissues (T/N ≥ 2) (Fig. 1b). The protein level of NRIP3 was upregulated in most ESCC cell lines compared to the nontumor tissue pool (composed of ten nontumor tissues) (Figs. 1c and S1B).

We also detected the expression of NRIP3 using an ESCC tissue microarray (TMA) (300 ESCC cases collected at Linzhou Cancer Hospital, Henan) and 73 ESCC cases (collected at SYSUCC, Guangzhou) by immunohistochemistry (IHC) staining. Samples from patients who did not have complete information were excluded from statistical analysis. In 273 informative paired tissues, NRIP3 upregulation was detected in 176 (64.47%) tumor tissues compared to paired nontumor tissues (*P* < 0.05). The overall survival curve was plotted by Kaplan–Meier analysis, and the difference in survival time was evaluated by the log-rank test. The median survival time of patients with upregulated NRIP3 was 35 months, which was significantly lower than the 46-month survival time of patients with normal NRIP3 expression (*P* < 0.001, Fig. 1d, e). The correlation between the upregulation of NRIP3 and the clinicopathological features of ESCC tissue samples was analyzed. NRIP3 upregulation was significantly correlated with tumor invasion (*P* = 0.003), lymph node metastasis (*P* < 0.001) and TNM staging (*P* < 0.001) (Table 1).

**Table 1:** Association analysis of NRIP3 upregulation in tumors with clinicopathologic characteristics of ESCC patients.

Clinicopathologic characteristics	Total	NRIP3 upregulation	*P* value
Sex			0.768
Female	113	74 (65.5%)	
Male	160	102 (63.8%)	
Age			0.948
<60	140	90 (64.3%)	
⩾60	133	86 (64.7%)	
Differentiation			0.834
Well	70	47 (67.1%)	
Moderate	134	86 (64.2%)	
Poor	69	43 (62.3%)	
Tumor invasion			**0.003**
0–1	82	41 (50.0%)	
2	69	46 (66.7%)	
3	122	89 (73.0%)	
LN metastasis			**<0.001**
−	203	116 (57.1%)	
+	70	60 (85.7%)	
TNM stage			**<0.001**
Early (0 + I)	78	41 (52.6%)	
Moderate (II + III)	150	94 (62.7%)	
Advanced (IV)	45	41 (91.1%)	

Bold values indicates statistical significance.

The relationship between NRIP3 upregulation and the prognosis of esophageal cancer was further analyzed by univariate analysis. NRIP3 upregulation, tumor invasion, lymph node metastasis and TNM staging were significantly associated with overall survival (*P* < 0.001), while there was no significant correlation between sex, age, and tumor differentiation and overall survival time. To rule out the possibility that each of the above variables was a covariate, further multivariate analysis was performed, and upregulation of NRIP3 was an independent prognostic factor for overall survival in ESCC (*P* = 0.005, Table 2).

**Table 2:** Univariate and multivariate analyses of prognostic variables in ESCC patients.

Clinicopathologic characteristics	Univariate analysis^a^		Multivariate analysis^a^	
	HR (95% CI)	*P* value	HR (95% CI)	*P* value
Sex	0.971 (0.694–1.359)	0.865		
Age	1.123 (0.807–1.562)	0.491		
Differentiation	1.197 (0.950–1.508)	0.128		
Tumor invasion	2.096 (1.677–2.620)	**<0.001**	1.224 (0.825–1.817)	0.315
LN metastasis	2.327 (1.647–3.286)	**<0.001**	0.714 (0.376–1.355)	0.303
TNM stage	2.712 (2.105–3.495)	**<0.001**	2.493 (1.275–4.874)	**0.008**
NRIP3 upregulation	2.301 (1.557–3.402)	**<0.001**	1.787 (1.191–2.680)	**0.005**

*HR* hazard ratio, *CI* confidence interval.

^a^Cox regression model was used in the analyses.

Bold values indicates statistical significance.

### NRIP3 facilitates ESCC tumor cell proliferation in vitro and in vivo

**Fig. 2:**
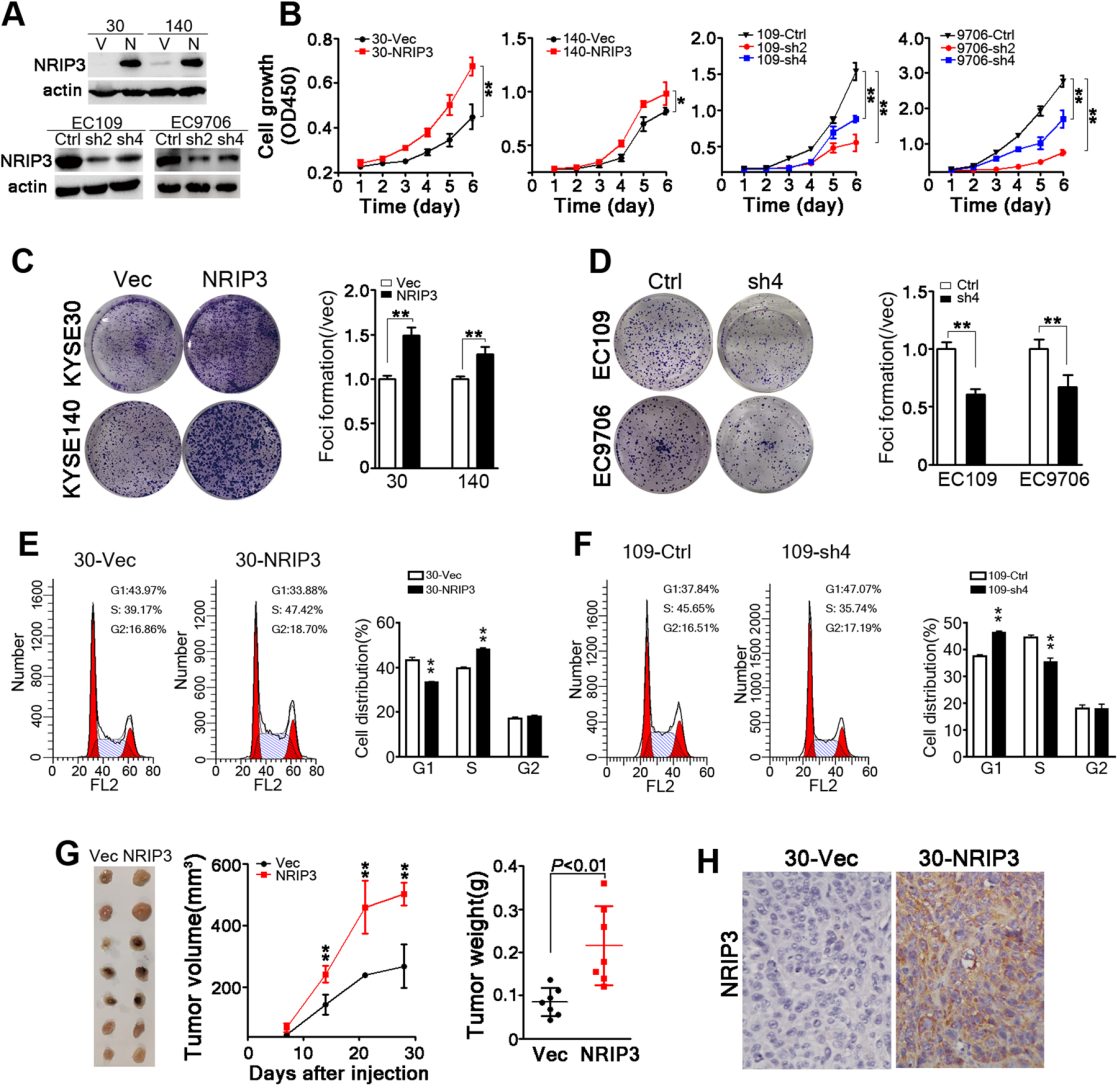
NRIP3 is functionally critical for ESCC tumor cell growth. **a** Western blotting analysis of NRIP3 expression in the indicated cell lines (V vector; N NRIP3). β-actin was used as a loading control. **b** The cell growth rate was evaluated in NRIP3-overexpressing cells (KYSE30 and KYSE140) and NRIP3-KD cells (EC109 and EC9706) (*P < 0.05; **P < 0.01). **c** Representative images and summary of foci formation in NRIP3-overexpressing cells (KYSE30 and KYSE140) (**P < 0.01). **d** Representative pictures and summary of foci formation in NRIP3-KD cells (EC109 and EC9706). Cell cycle was analyzed by flow cytometry in NRIP3-overexpressing cells (**e**) and NRIP3-KD (**f**) cells (left: representative images; right: summary). **g** The image of xenografts formed in the nude mice (left), tumor growth curve (middle) and tumor weight (right) are summarized (**P < 0.01). **h** Representative IHC staining images comparing NRIP3 expression between 30-Vec and 30-NRIP3 xenografts (original magnification: ×200).

To evaluate the functional impact of NRIP3 in ESCC tumor cells, NRIP3 was overexpressed in KYSE30 and KYSE140 cells that have relative low level of NRIP3 (Fig. 2a). In addition, endogenous NRIP3 was knocked down using lentivirus containing shRNAs targeting NRIP3 in EC109 and EC9706 cells (Fig. 2a). The cell proliferation assay results revealed that NRIP3 overexpression increased cell proliferation, while silencing NRIP3 decreased cell growth (Fig. 2b). Anchorage-dependent colony formation assays were performed, and the results indicated that colony formation increased significantly in NRIP3-overexpressing cells compared with vector control cells, and vice versa (Fig. 2c, d). Flow cytometry results indicated that NRIP3-overexpressing cells (30-NRIP3) exhibited a decrease in the G1 phase population and an increase in the S phase population. When NRIP3 was knocked down, the cells were arrested at the G1/S checkpoint (Fig. 2e, f). In line with the results, 5-Ethynyl-2′-deoxyuridine (EdU) positive cells were increased in NRIP3-overexpressing cells and decreased in NRIP3-KD cells (Fig. S2). In vivo xenograft formation assay results revealed that xenograft growth and weight increased significantly in NRIP3-overexpressing KYSE30 cells (*P* < 0.01, Fig. 2g, h). When NRIP3 was silenced in EC9706 cells with shRNAs, tumor growth and weight decreased compared with those in control cells (Fig. S3). In aggregate, our results demonstrate that NRIP3 overexpression facilitates ESCC tumor cell proliferation.

RNA sequencing was performed on EC109-sh2 and control cells and gene expression profiling revealed that the enrichment of lipid metabolism was observed in NRIP3-silenced EC109 cells compared with control cells (Fig. S4). Next, we performed the nontarget lipid mass spectrometry analysis on 30-NRIP3 cells and EC109-KD cells and their corresponding control cells. As Fig. S5 shows, among the 71 classes of lipids detected, 7 classes of lipids were identified as significant based on the similarities in the results of the overexpression group (30-NRIP3 versus 30-Vec) and the knockdown group (EC109-sh4 versus EC109-Ctrl). Notably, NRIP3-overexpressing cells displayed reduced ceramide (Cer) levels by up to 50% (Fig. S5A). When endogenous NRIP3 was silenced in EC109 cells, the Cer level increased significantly (Fig. S5A).

### NRIP3 protects ESCC tumor cells from DNA damage

**Fig. 3:**
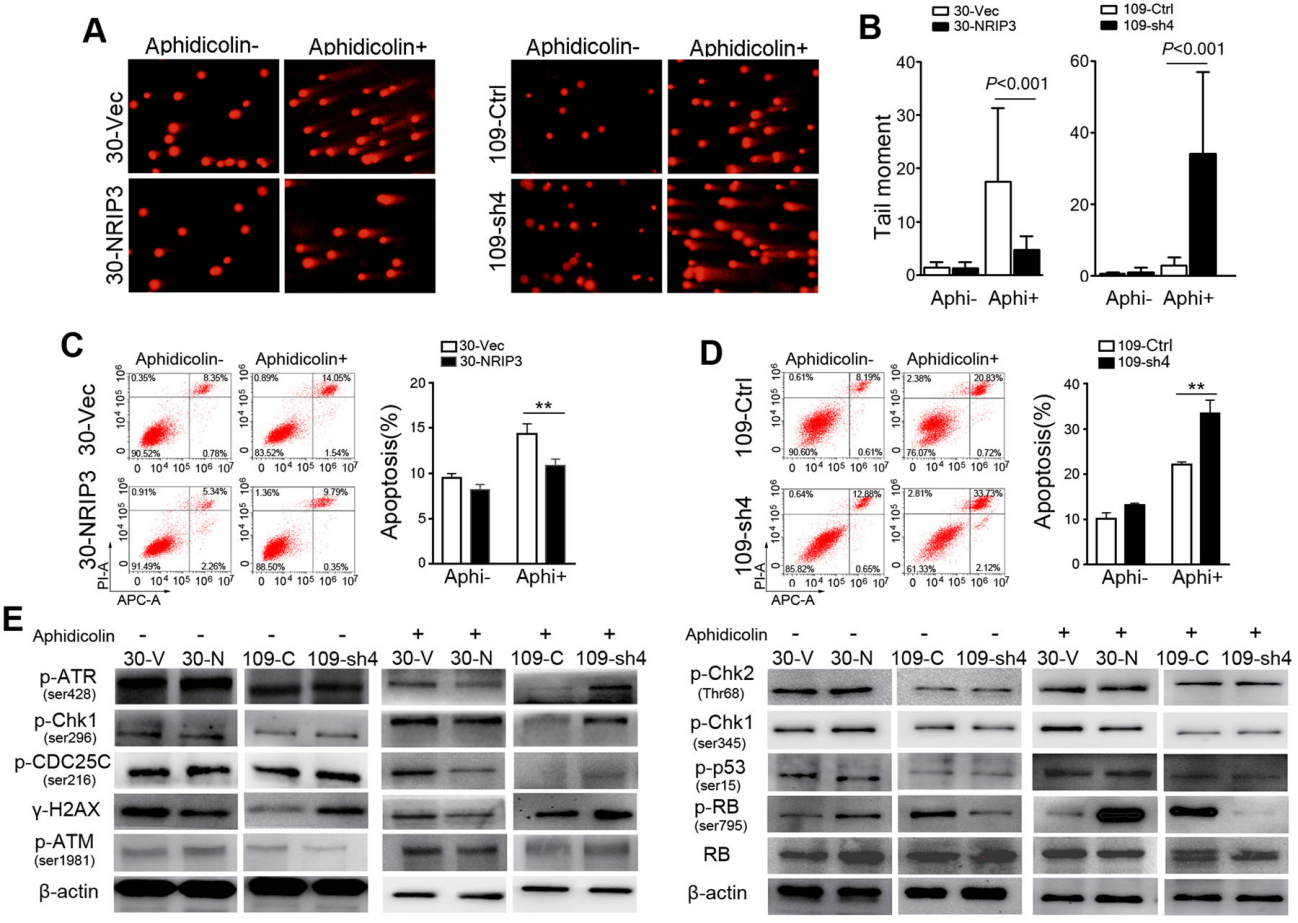
NRIP3 induces protection against DNA damage. **a**, **b** DNA damage assessed by an alkaline comet assay in cells treated with or without aphidicolin. Representative pictures (**a**); summary (**b**). Apoptosis assessed by flow cytometry in NRIP3-overexpressing cells (**c**) and NRIP3-KD cells (**d**) treated with or without aphidicolin (**P < 0.01). **e** Immunoblotting was performed using total protein lysates from NRIP3-overexpressing cells or -KD cells treated with or without aphidicolin.

Because Cer is directly involved in the interlinked signaling pathways underlying the cellular DNA damage response to chemotherapy and ionizing radiation (IR)^19,20^, we wondered whether NRIP3 could protect cells from DNA damage. The comet assay indicated that the NRIP3-overexpressing cells exhibited resistance to treatment with aphidicolin, an inhibitor of the replicative polymerase^21^ (Fig. 3a, b). Consistently, NRIP3-KD cells exhibited sensitivity to aphidicolin (Fig. 3a, b). We next examined the apoptosis rate following aphidicolin treatment in NRIP3-overexpressing and -KD cells. The apoptosis rate was significantly ameliorated in NRIP3-overexpressing cells compared to control cells after aphidicolin treatment (*P* < 0.01, Fig. 3c). In contrast, NRIP3-KD cells exhibited an increase in the apoptosis rate after aphidicolin treatment (*P* < 0.01, Fig. 3d). These results collectively demonstrate that NRIP3 can protect ESCC cells during replication stress.

We next investigated damage signaling in cells with NRIP3 overexpression or knockdown. NRIP3-KD cells showed elevated levels of phosphorylation of ATR (Ser428), Chk1 (Ser296), Cdc25C (Ser216), and γH2AX as well as decreased Rb phosphorylation (Ser795) following aphidicolin treatment. Likewise, overexpression of NRIP3 suppressed the phosphorylation of ATR (Ser428), Chk1 (Ser296), and Cdc25C (Ser16) and γH2AX as well as increased Rb phosphorylation (Ser795) in KYSE30 cells following aphidicolin treatment (Fig. 3e).

### NRIP3 accelerates RTF2 removal via interacting with DDI1 and RTF2

**Fig. 4:**
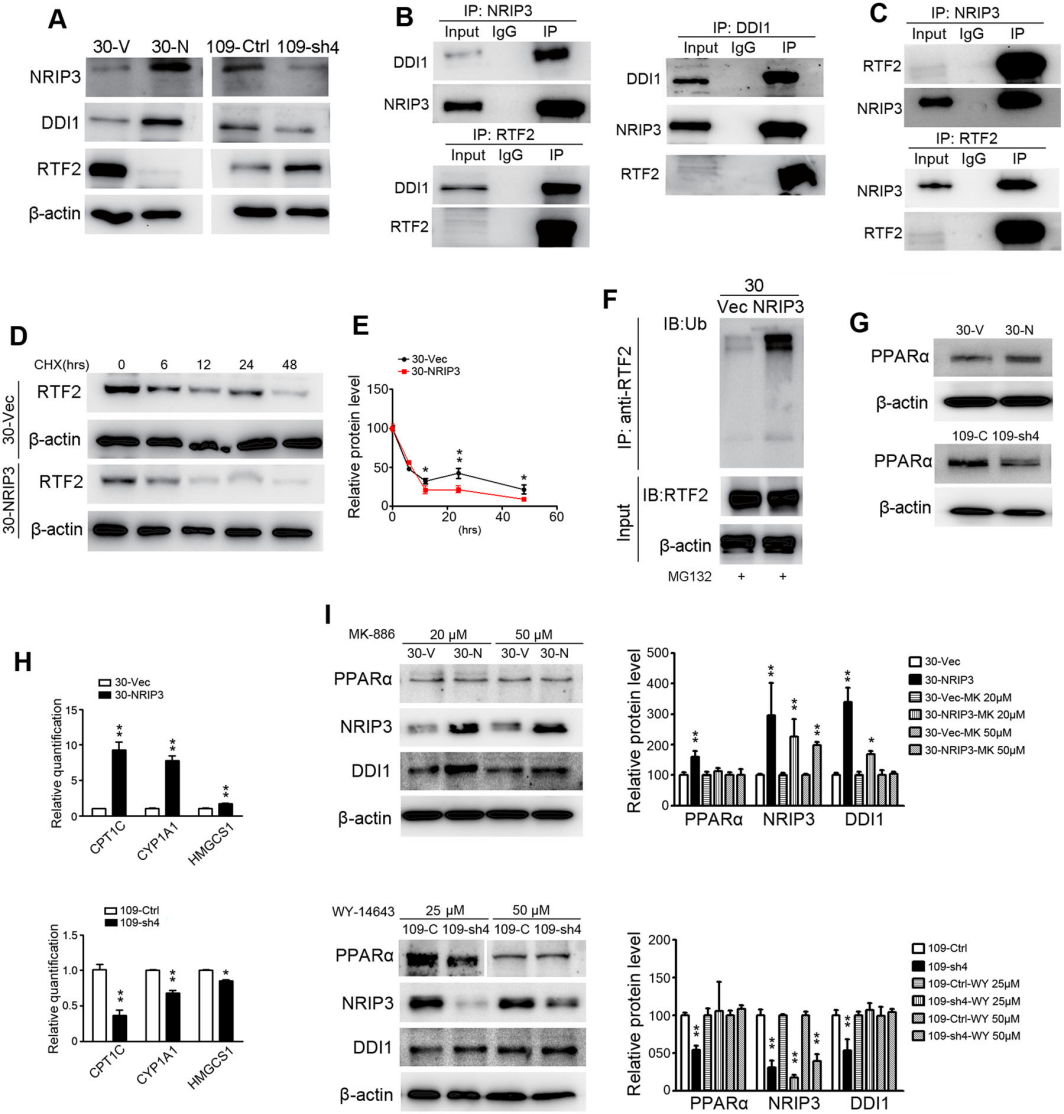
NRIP3 accelerates RTF2 removal. **a** Protein levels of NRIP3, DDI1, and RTF2 in 30-NRIP3 (30-N), EC109-KD, and the respective control cells. **b** Co-IP experiments were performed in 30-NRIP3 cells using NRIP3 antibody, DDI1 antibody, or RTF2 antibody to pull-down proteins, and the pull-down proteins were then detected by western blotting. **c** Co-IP experiments were performed in EC109 cells using NRIP3 or RTF2 antibodies, and the pull-down proteins were detected by western blotting. **d**, **e** CHX chase experiments demonstrated the degradation of RTF2 protein in 30-NRIP3 and 30-Vec cells. Western blotting data were quantified in (**e**). Data are presented as the mean ± SEM. **f** 30-NRIP3 and 30-Vec cells were subjected to IP with RTF2 antibody. Polyubiquitylated RTF2 protein was visualized by ubiquitin antibody. **g** Western blotting analysis of PPARα in 30-NRIP3 and EC109-KD cells compared with the respective control cells. **h** qRT-PCR analysis of the expression levels of three PPARα target genes (CPT1C, CYP1A1, and HMGCS1) in NRIP3-overexpressing and -KD cells (**P < 0.01). **i** Western blotting analysis of PPARα, NRIP3, and DDI1 in the 30-NRIP3 cells treated with MK-886 (PPARα antagonist) or 109-KD cells treated with WY-14643 (PPARα agonist).

By searching the BioGRID database (https://thebiogrid.org/) and GPS-Prot Interaction Network, a protein named DDI1 (DNA-damage inducible 1 homolog 1) was screened out as a potential candidate interacting with NRIP3^22,23^ (Table S1). Western blotting analysis revealed an upregulation of DDI1 in NRIP3-overexpressing cells (30-NRIP3) and a downregulation of DDI1 in NRIP3-KD cells (109-sh4) (Fig. 4a).

Coimmunoprecipitation (Co-IP) experiments were performed in 30-NRIP3 cells. Consistent with the high-throughput analysis results^22,23^, co-IP results demonstrated that NRIP3 could interact with DDI1. This interaction was confirmed by co-IP experiments for DDI1 protein using NRIP3 antibody and then by reciprocal IP of the NRIP3 protein with DDI1 antibody (Fig. 4b). According to a recent report^18^, removal of an RTF2 is a critical step during response to replication stress. DDI1 is a proteasomal shuttle protein, which is required for RTF2 removal for replication fork recovery from replication stress. The interaction between DDI1 and RTF2 was also confirmed by co-IP experiments (Fig. 4b). Since RTF2 decreased in NRIP3-overexpressing cells, we used EC109 cells to explore whether there was an interaction between NRIP3 and RTF2. Interestingly, we found that NRIP3 could interact with RTF2 using co-IP experiments (Fig. 4c).

Since RTF2 decreased in NRIP3-overexpressing cells and increased in NRIP3-KD cells (Fig. 4a), we blocked protein synthesis using cycloheximide (CHX) and chased the RTF2 protein level in the 30-NRIP3 and vector control cells. Indeed, RTF2 was degraded rapidly in 30-NRIP3 cells with CHX treatment (Fig. 4d, e). Furthermore, 30-NRIP3 and control cells were treated with the proteasomal inhibitor MG-132 for 12 h to prevent protein degradation before performing an ubiquitylation assay. A significant increase in polyubiquitinated RTF2 protein was observed in 30-NRIP3 cells compared with vector control cells (Fig. 4f). These results indicate that proteasome-mediated degradation is the key contributor to the stability of RTF2.

Because gene set enrichment analysis revealed that NRIP3 knockdown resulted in the enrichment of genes related to the PPARα pathway (Fig. S4), we wondered whether PPARα was involved in DDI1 protein level alterations in NRIP3-overexpressing or knockdown cells. The protein level of PPARα increased in NRIP3-overexpressing cells and decreased in NRIP3-KD cells (Fig. 4g). Some genes involved in the PPARα pathway, CPT1C^24^, CYP1A1^25^, and HMGCS1^26^, were detected by qRT-PCR. The results demonstrated elevated expression of the PPARα target genes in 30-NRIP3 cells and decreased expression of those genes in 109-sh4 cells (Fig. 4h), confirming PPARα activation in NRIP3-overexpressing cells and inactivation in knockdown cells. To directly evaluate the functional importance of PPARα in DDI1 upregulation in NRIP3-overexpressing cells, a PPARα antagonist, MK-886, was added to 30-NRIP3 and control cells. The increase in DDI1 protein levels was attenuated by the increase in MK-886 concentration (Fig. 4i). Consistently, EC109-sh4 cells were treated with a PPARα agonist, WY-14643, and the decrease in DDI1 was abrogated by the treatment (Fig. 4i). Overall, these findings indicate that NRIP3 manipulates the expression of DDI1 at least partly via the PPARα pathway.

### NRIP3 confers resistance to chemoradiotherapy in ESCC cells

**Fig. 5:**
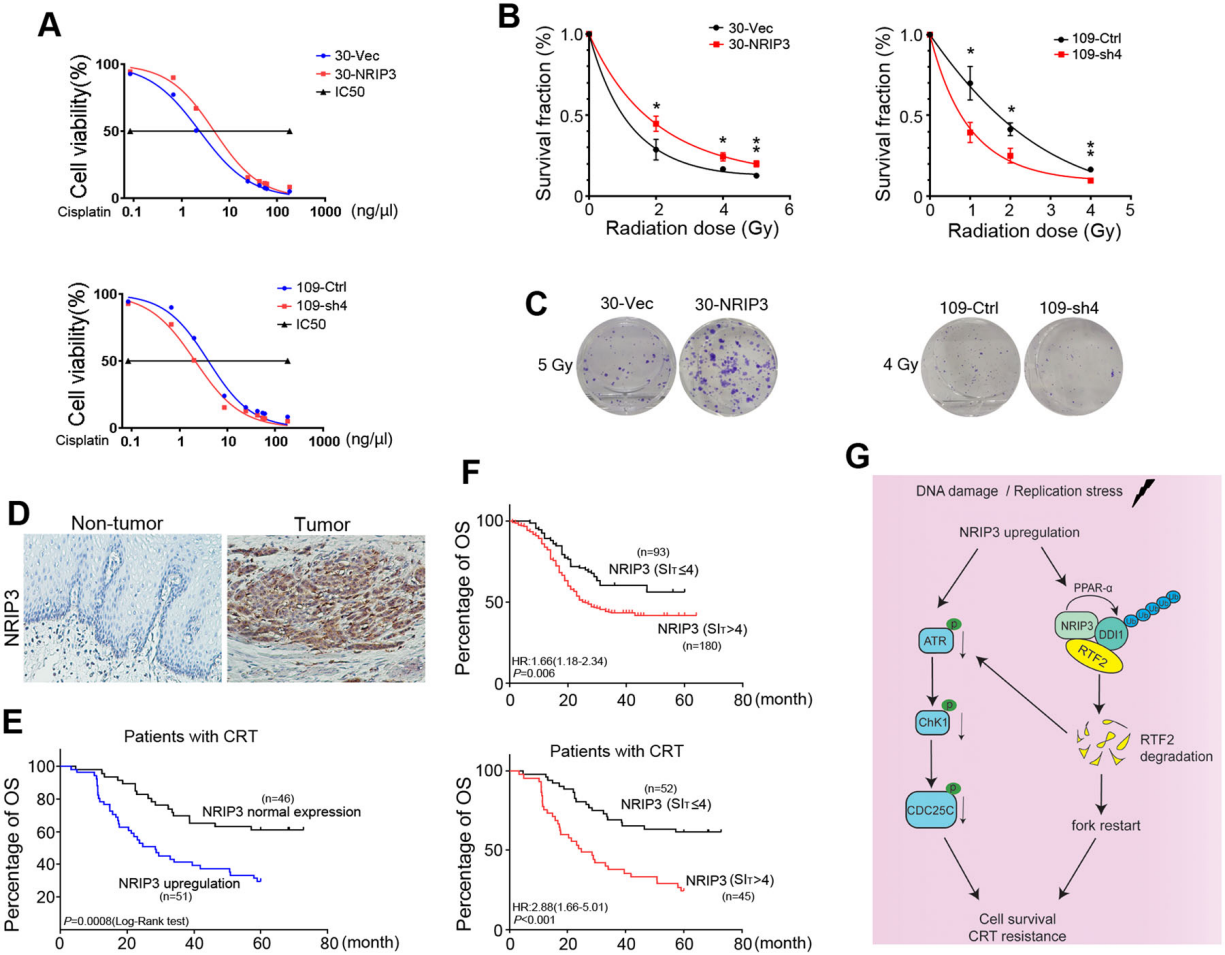
NRIP3 upregulation confers resistance to chemoradiotherapy in ESCC. **a** NRIP3-overexpressing and NRIP3-KD cells were treated with cisplatin, and cell viability was determined by cell growth assay. **b** Clonogenic survival analysis of 30-NRIP3 and 109-KD and the respective control cells (*n* = 3, *P < 0.05; **P < 0.01). **c** Representative images of cell colony formation after a radiation dose of 5 Gy for 30-NRIP3 and control cells, and 4 Gy for 109-KD and control cells. **d** Representative IHC images of NRIP3 in ESCC tumor tissues and paired nontumor tissues of patients receiving chemoradiotherapy (original magnification: ×200). **e** Overall survival analysis of the NRIP3 upregulation signature (SI_T_ > SI_N_) in ESCC patients receiving cisplatin-based chemotherapy and/or radiotherapy (Kaplan–Meier analysis). **f** Overall survival analysis of the NRIP3 upregulation signature (SI_T_ > 4) in patients with ESCC receiving CRT or not (Kaplan–Meier analysis) (top, without CRT; bottom, with CRT) (hazard ratio was calculated using log-rank test). **g** Schematic model of the functional role of the NRIP3-PPARα-DDI1-RTF2 axis in conferring resistance to replicative stress in ESCC.

We further examined whether NRIP3 overexpression could increase drug resistance to cisplatin since it is a widely used chemotherapeutic drug that causes DNA damage in cancer cells^27^. Cell growth assays revealed that the resistance to cisplatin in NRIP3-overexpressing KYSE30 cells was significantly increased, with a twofold increase in half maximal inhibitory concentration (IC50) value compared to that in control cells (*P* < 0.05, Fig. 5a). The similar results were also observed in NRIP3-overexpressing KYSE150 and KYSE140 cells (Fig. S6A, B). In line with the results, compared with the control cells, NRIP3-KD cells exhibited increased sensitivity to cisplatin (*P* < 0.05, Fig. 5a). In addition, we found that NRIP3-overexpressing cells exhibited resistance to carboplatin, another commonly used platinum salt, compared to vector control cells (Fig. S6C). For paclitaxel, no significant resistance was observed in NRIP3-overexpressing cells (Fig. S6D).

Response to radiotherapy was investigated in a colony-forming assay. The survival fraction (SF) after treatment with a radiation dose of 5 Gy was SF_5GY_ = 0.20 for 30-NRIP3 and SF_5GY_ = 0.12 for control cells (*P* < 0.01, Fig. 5b). The SFs of NRIP3-KD cells and control cells also differed significantly, as shown in Fig. 5b (SF_4GY_ = 0.09 for 109-sh4 and SF_4GY_ = 0.16 for 109-Ctrl) (*P* < 0.01, Fig. 5b, c). Comet assay results showed that NRIP3 overexpression could protect cells from DNA damage induced by cisplatin or IR (Fig. S7A). Consistently, tail moment increased in NRIP3-KD cells treated with cisplatin or IR (Fig. S7B). In addition, western blotting results showed significant changes of phosphorylation of ATR (Ser428), Cdc25C (Ser216), Chk1 (Ser345 and Ser296), and γH2AX in NRIP3-KD cells when cells were treated with IR (Fig. S8). To evaluate the effect of combination treatment, cells were treated with chemotherapeutic drug (cisplatin or carboplatin) and IR. NRIP3-overexpressing cells exhibited resistance to cisplatin/carboplatin combined with IR (Fig. S9). Taken together, these data suggest that NRIP3 overexpression leads to chemoresistance to cisplatin and radioresistance in ESCC cells.

### NRIP3 upregulation predicts a poorer prognosis in ESCC patients receiving chemoradiotherapy

To investigate whether the CRT resistance effects obtained in the in vitro experiments would translate into a clinical setting, the clinical significance of NRIP3-related chemoradioresistance in ESCC was explored. We collected ESCC tumor tissues and paired nontumor tissues from patients receiving cisplatin-based chemotherapy or radiotherapy after surgery in SYSUCC. We then stratified patients with ESCC by NRIP3 expression to evaluate responses to CRT. The IHC results demonstrated that in 97 informative cases, 51 cases exhibited NRIP3 upregulation compared with nontumor tissues (Fig. 5d). NRIP3 upregulation was related to a poor prognosis in terms of overall survival (*P* < 0.001, log-rank test) (Fig. 5e), with the 5-year survival rate being 29.4% (16.85–41.94%) in the NRIP3 upregulation group and 60.9% (46.79–75%) in the NRIP3 normal expression group. These results highlight the clinical relevance of chemoradioresistance induced by NRIP3 upregulation in patients with ESCC.

To further extend the clinical significance of NRIP3 upregualtion in treatment regimen options for ESCC, we subjected the NRIP3 level data to ROC curve analysis with respect to overall survival, and the cutoff value for NRIP3 was 4. NRIP3 upregulation was determined as IHC staining index (SI) > 4. As shown in Fig. 5f, NRIP3 upregualtion (SI > 4) has a poor prognosis value for overall survival in patients with ESCC.

## Discussion

In this study, we identified an NRIP3-mediated pathway that facilitates resistance to replication stress induced by cisplatin-based chemotherapy or radiotherapy. As one of the nuclear receptor interacting proteins, NRIP3 has been reported to form a transforming *MLL-NRIP3* gene in acute leukemia and to correlate with the prognosis of breast cancer by bioinformatics analysis^12–14^. Our study suggests an important functional role of NRIP3 in promoting ESCC cell growth and conferring resistance to DNA damage by increasing DDI1 and accelerating RTF2 removal for replication fork recovery. NRIP3 upregulation is correlated with poor overall survival in patients with ESCC.

The nontarget lipid mass spectrometry analysis showed that NRIP3 overexpression induced decreases in Cer and TGs. Here, we provide evidence for an important functional role of NRIP3 in repressing Cer, a crucial sensor and/or effector of various signaling pathways promoting cell cycle arrest, apoptosis and differentiation^28^. Consistent with this finding, we observed decreased cell proliferation and G1/S arrest in NRIP3 knockdown cells.

Several studies have identified Cer elevation as a key mediator in the DNA damage response to IR^19,29^. The Cer decreased in NRIP3-overexpressing cells and it increased in NRIP3-KD cells, leading us to wonder whether NRIP3 could protect cells from DNA damage. DNA is constantly damaged by endogenous and exogenous stimuli. Endogenous lesions include incomplete DNA replication due to stalled replication forks and reactive oxygen species, while exogenous influences are exemplified by chemical or physical factors, such as DNA damaging chemotherapeutics, UV light, and IR^30^. Our results showed that DNA damage was ameliorated in NRIP3-overexpressing cells when cells were treated with aphidicolin (an inhibitor of replicative polymerase), cisplatin and IR, suggesting that NRIP3 confers upon ESCC cell resistance to replicative stress and CRT. Previous studies suggest that 1 Gy of IR will generate ~1000 SSBs (single-strand breaks) and 35 DSBs (double-strand breaks) in human cells^31^. Cisplatin is one of platinum salts, which are commonly used in DNA-damage-inducing chemotherapies in many cancers. It generates covalent cross-links between DNA bases^32^. Based on the previous studies and our observations, we believe that the relevant DNA damage types in the context of NRIP3 manipulations are: SSBs, DSBs, and covalent cross-links between DNA bases.

To explore the mechanism of NRIP3 resistance to DNA damage, we screened bioinformatics databases and identified that NRIP3 could interact with DDI1, a proteasomal shuttle protein participating in the cellular response to DNA replication stress^33^. This is consistent with the high-throughput protein interaction analyses^22,23^. DDI1 functions by virtue of modular ubiquitin-like and ubiquitin-associated domains, allowing them to interact with both the proteasome and ubiquitinated substrates^34,35^. DDI1 is known to be critical for removal of a replisome component RTF2 (also known as C20orf43) from stalled forks during proper response to replication stress^18^. Our data show that NRIP3 accelerates RTF2 ubiquitylation degradation by increasing the protein level of DDI1 and interacting with DDI1 and RTF2. This is also consistent with our results showing decreased DNA damage in NRIP3-overexpressing cells following aphidicolin treatment.

Exploring the mechanism by which NRIP3 induces an increase in the protein level of DDI1, we detected a decrease in PPARα in NRIP3-KD cells and an increase in PPARα in NRIP3-overexpressing cells. PPARα is one of the different subtypes of PPARs^36^. It is a nuclear receptor, and it functions as a ligand-activated transcription factor responsible for the induction of the majority of genes necessary for fatty acid transport, uptake, and oxidation, as well as ketone metabolism^37,38^. We found that the decrease in DDI1 was attenuated in NRIP3-KD cells treated with a PPARα agonist. Consistent with this, the increase in DDI1 was alleviated in NRIP3-overexpressing cells treated with a PPARα antagonist. Taken together, these results indicate that the activated PPARα pathway is a critical mechanism by which the DDI1 protein level is increased.

In conclusion, our study reveals a functionally important novel mechanism of NRIP3 upregulation in ESCC progression and CRT resistance. Our results suggest a model that NRIP3 upregulation increases DDI1 at least partly via PPARα; NRIP3, DDI1, and RTF2 form a complex and facilitate RTF2 removal by proteosomal degradation of RTF2, thereby restarting the replication fork when cells are in replicative stress (Fig. 5g). RTF2 has been identified to promote single-stranded DNA accumulation and ATR activation during replication stress^18^. We also note significant decreased signaling of the ATR/Chk1pathway in NRIP3-overexpressing cells when cells are treated with IR. These observations suggest that both mechanisms interact together in ESCC cells acquiring CRT resistance (Fig. 5g).

The importance of investigating the resistance of NRIP3 to DNA damage is further strengthened by the finding of increased resistance to platinum salts (cisplatin, carboplatin)-based chemotherapy and radiation in NRIP3-overexpressing cells. The mode of action of platinum salts has been linked to its ability to crosslink with purine bases on DNA, interfering with DNA repair mechanisms and causing DNA damage^27^. Since platinum-based chemotherapy and radiotherapy have been applied in ESCC treatment regimens, patients with ESCC who underwent postoperative cisplatin-based chemotherapy or radiotherapy were screened, and overall survival analysis was carried out. Our findings demonstrate that NRIP3 upregulation predicts a poor prognosis in ESCC patients with chemotherapy and/or radiotherapy. The survival analysis also confirmed a positive correlation between NRIP3 upregulation and resistance to CRT. To extend the clinical significance of NRIP3 in nCRT, which is commonly used in ESCC, we subjected the NRIP3 expression level to ROC curve analysis and set the SI > 4 as NRIP3 upregulation. Future studies will be necessary to investigate NRIP3 expression in the biopsies of the patients receiving nCRT.

Taken together, NRIP3 is upregulated in ESCC tumor tissues. Our study suggests that NRIP3-PPARα-DDI1-RTF2 is a novel protective mechanism in ESCC that mediates resistance to replication stress signals. NRIP3 upregulation confers resistance to cisplatin, carboplatin, and IR. NRIP3 is a prognostic factor in ESCC and could have some predictive value to select patients who benefit from CRT treatment.

## Materials and methods

### Tissue microarray (TMA), tissues, and immunohistochemistry (IHC)

A total of 300 ESCC patient tissues (including primary tumor tissues and paired nontumor tissues) were collected from Linzhou Cancer Hospital (Henan Province, China). Seventy-three cases of ESCC tumor and paired nontumor tissues were collected at Sun Yat-sen University Cancer Center (SYSUCC, Guangzhou, China). Patients recruited in this study did not receive any preoperative treatment. The research was approved by the Institutional Review Boards (IRBs) of SYSUCC and Zhengzhou University. Written consent from the patients was acquired. The TMA was constructed, and immunohistochemical staining was performed according to a previous report^39^. Briefly, the paraffin-embedded TMA sections were deparaffinized and rehydrated with xylene and a series of ethanol concentrations. After antigen retrieval, the sections were incubated with 3% hydrogen peroxide. Next, the sections were blocked and incubated with NRIP3 antibody (1:600; Novus Biologicals, Littleton, CO) overnight at 4 °C. Two investigators scored the degree of immunostaining independently. A SI (0–12) was calculated by multiplying the staining intensity (negative—0; weak—1; moderate—2; or strong—3) by the percentage of NRIP3-positive staining (<5%—0; 5–25%—1; 25–50%—2; 50–75%—3; >75%—4). The upregulation of NRIP3 was defined when the SI of tumor tissue was higher than that of corresponding nontumor tissue. Samples that were dropped and lacked representative samples were excluded from the analysis.

IHC was also performed on 105 pairs of ESCC tumor tissues and nontumor tissues collected from SYSUCC. These patients received radiotherapy or cisplatin-based chemotherapy after resection (Table S2). The patients’ ages ranged from 37 to 79. The male/female ratio was 7.75. The research was approved by the IRB of SYSUCC. Written consent from the patients was acquired.

Forty pairs of ESCC tumor tissues and adjacent nontumor tissues were obtained from Linzhou Cancer Hospital for RNA extraction. The study was approved by the IRBs of Sun Yat-sen University Cancer Center and Zhengzhou University. Written consent from the patients was acquired.

### Cell lines

HKESC1, EC18, EC109, and EC9706 were kindly provided by professor G. Srivastava and professor G.S. Tsao of the University of Hong Kong^40^. KYSE30, KYSE140, KYSE150, KYSE180, KYSE410, KYSE510, and KYSE520 were acquired from DSMZ (the German Resource Center for Biological Material)^41,42^. 293FT was purchased from Invitrogen (Carlsbad, CA). The ESCC cells were authenticated by STR profiling and mycoplasma was tested negative.

### DNA comet assay

Cells were treated with aphidicolin (0.4 μM for KYSE30, 0.2 μM for EC109), or cisplatin (3 μg/ml for KYSE30, 2 μg/ml for EC109) for 24 h, or exposed to 4 Gy radiation (X-ray irradiator, RS2000, Rad Source, USA). A total of 15–20 µl cell suspension was mixed with 80 µl low-melting agarose gel, spread onto comet slides, allowed to set at 4 °C for 10 min and lysed at 4 °C for at least 1 h. After washing with PBS, slides were placed in the electrophoresis solution to allow for unwinding of the DNA and the expression of alkali-labile damage. Electrophoresis was then conducted at 25 V for 15–20 min in the electrophoresis solution. The slides were neutralized, washed, stained with PI (Sigma-Aldrich, Saint Louis, MO), and finally observed under a fluorescence microscope (Eclipse 90i; Nikon, Tokyo, Japan), and the DNA tail percentage was analyzed using the Casp software program (version 1.2.3) (CASPLab Comet Assay Project).

### Coimmunoprecipitation assay and western blot analysis

Co-IP assays were performed using a Pierce Direct Magnetic IP/Co-IP kit (Thermo Scientific, Rockford, IL) according to the manual. Briefly, cells were incubated with lysis buffer on ice for 5 min with periodic mixing. The lysis solution was quantified and diluted. Five hundred microliters of lysate was then incubated with 25 µl of beads coupled with 5 µg of antibody at 4 °C overnight. After extensive washing, the beads were spun down, resuspended and boiled. The pull-down samples were detected using western blotting according to standard protocols.

### Ubiquitination assay

Cells were treated with 10 μM MG-132 for 12 h to block proteosomal degradation. Cell lysates were collected and immunoprecipitated with anti-RTF2 antibody (5 µg), eluted with 20 μL lysis buffer and denatured with SDS sample buffer (Thermo Scientific, Rockford, IL). Pull-down samples were subjected to immunoblotting with an antiubiquitin antibody to visualize polyubiquitylated RTF2 protein bands.

### Apoptosis assay

Cells were treated with aphidicolin (0.4 µM for KYSE30 and 0.2 µM for EC109) for 24 h. Cells were then harvested and stained using an annexin V/PI apoptosis kit (Best Bio, Shanghai, China). Cell samples were analyzed by CytoFLEX (Beckman Coulter, Fullerton, CA). Three independent assays were performed.

### Cell cycle analysis and EdU assay

KYSE30 and EC109 cells (5 × 10^5^) were fixed in precooled 75% ethanol at 4 °C for 2 h. Cells were incubated with RNase to remove RNA at 37 °C for 30 min, and then stained with propidium iodide (Sigma-Aldrich, Saint Louis, MO). The samples were analyzed by CytoFLEX (Beckman Coulter, Fullerton, CA). Cell cycle analysis was performed by Modfit LT 3.1 (Beckman Coulter). Three independent assays were repeated.

The cells were incubated with EdU (RiBoBio, China) for 4 h (KYSE30: 10 µM EdU; EC109: 20 µM EdU). EdU positive cells were analyzed by CytoFLEX. Three independent assays were repeated.

### Statistical analyses

All data are shown as the mean ± SEM (standard error of the mean). Statistical comparisons were performed using SPSS 20.0 software (SPSS, Inc., Chicago, IL). The Kaplan–Meier method was used for survival analysis, and statistical analysis was performed using the log-rank test. Pearson’s and *χ*^2^ tests were used to examine the correlation between NRIP3 expression and clinicopathological features. A Cox proportional hazard regression model was used to assess independent prognostic factors. Student’s *t* test was performed to compare the differences between two groups of samples. *P* < 0.05 was regarded as statistically significant.

Supplementary information about “Materials and methods” is provided in the Supporting information.

## Supplementary information

Supporting information

Figure S1

Figure S2

Figure S3

Figure S4

Figure S5

Figure S6

Figure S7

Figure S8

Figure S9
