# Supplementary material for: NRIP3 upregulation confers resistance to chemoradiotherapy in ESCC via RTF2 removal by accelerating ubiquitination and degradation of RTF2
Source: Oncogenesis. 2020 Aug 24;9(8):75. doi: 10.1038/s41389-020-00260-4 (PMC7445249; doi:10.1038/s41389-020-00260-4)
Supplement: Supplementary file 1 — Supporting information [file 41389_2020_260_MOESM1_ESM.docx]

**Supplemental information:**

**NRIP3 upregulation confers resistance to chemoradiotherapy in ESCC via RTF2 removal by accelerating ubiquitination and degradation of RTF2**

Daqin Suo^1#^, Ling Wang^1#^, Tingting Zeng, Hui Zhang^1,2^, Lei Li^1,3^, Jinyun Liu^1,2^, Jingping Yun^1^, Xin-Yuan Guan^1,3*^, Yan Li^1*^

**Supplementary materials:**

**Primers and shRNAs:**

| Primers: |  |
| --- | --- |
| NRIP3-F1 | 5’‐GGGCCTCTAAGACGAACAAAC‐3’ |
| NRIP3-R1 | 5’‐GCCTGTGTCAACCAAGGCT‐3’ |
| β-actin-F1 | 5’‐CATGTACGTTGCTATCCAGGC‐3’ |
| β-actin-R1 | 5’‐CTCCTTAATGTCACGCACGAT‐3’ |
| shRNAs targeting NRIP3: |  |
| sh2 | ggctgcctatataatctcatc |
| sh4 | ccttccatctaatagaagctc |

**Antibodies used in the study:**

| **Antibody** | **Company** | **Catalog number** |
| --- | --- | --- |
| NRIP3 | Novus Biologicals, | 45876 |
| NRIP3 | Gene Tex | 87860 |
| β-Actin | Cell Signaling Technology | 4970 |
| Phospho-ATR（S428） | Cell Signaling Technology | 2853 |
| Phospho-ATM（S1981） | Cell Signaling Technology | 5881 |
| Phospho-Chk1（S345） | Cell Signaling Technology | 2348 |
| Phospho-Chk1（S296） | Cell Signaling Technology | 2349 |
| Phospho-Chk2（T68） | Cell Signaling Technology | 2197 |
| Phospho-H2AX（S139） | Cell Signaling Technology | 9718 |
| Phospho-P53（S15） | Cell Signaling Technology | 9286 |
| Phospho-CDC25（S216） | Cell Signaling Technology | 47322 |
| Phospho-Rb (Ser795) | Cell Signaling Technology | 9301 |
| Rb | Cell Signaling Technology | 9309 |
| DDI1 | Gene Tex | 103772 |
| RTF2 | Proteintech | 166233-1-AP |
| PPAR-α | Gene Tex | 100539 |
| Rabbit anti-human IgG | Cell Signaling Technology | 7074 |
| Mouse anti-human IgG | Cell Signaling Technology | 7076 |

**Reagents used in the study:**

| **Reagent** | **Company** | **Catalog number** |
| --- | --- | --- |
| aphidicolin | Abcam | 142400 |
| cisplatin | Hansoh Pharma | 170402 |
| carboplatin | Bristol-Myers Squibb | 1C00723. |
| paclitaxel | LUMMY | Z161107 |
| MK-886 | Selleck | 8236 |
| WY-14643 | Selleck | 8029 |
| MG-132 | Selleck | 2619 |
| cycloheximide | Santa Cruz Biotechnology | 3508B |
| EdU | RiBoBio | C10338-3 |

**Supplementary materials and methods:**

ROC curve analysis:

We subjected the staining index scores of NRIP3 to ROC curve analysis (SPSS, Chicago, IL) with respect to overall survival and the optimal cutoff value was 4. NRIP3 upregulation in tumor tissues was then determined as IHC staining index >4.

**Plasmids, lentivuses, antibodies and reagents:**

Plasmid pEZ-LV105-NRIP3, OmicsLink^TM^ shRNA expression clones and Lenti-Pac™ HIV Expression Packaging Kit were purchased from GeneCopoeia (Guangzhou, China). 293FT cells were transfected with lentiviral plasmids and packaging kits following the manufacturer’s protocol to generate lentiviruses. Cells were transduced with lentiviruses and selected with the corresponding antibiotic resistance to establish cell lines with stable overexpression or knockdown. Antibodies and reagents are listed in the supplementary information.

**RNA sequencing and quantitative real-time polymerase chain reaction (qRT-PCR)**

Total RNA was extracted from EC109 derivative cells (EC109-sh2 and control) by using TRIzol reagent (Thermo Fisher Scientific, Waltham, MA) according to the manufacturer's instructions. RNA sequencing was conducted by Novogene (Hangzhou, China). Adjusted genes with *P* <0.05 found by edgeR were designated as differentially expressed. Gene set enrichment analysis (GSEA) was performed to identify the biological processes enriched in the experimental groups.

The extracted RNA was reverse-transcribed using EvoScript Universal cDNA Master (Roche, Basel, Switzerland). Real time PCR was carried out with the FastStart Universal SYBR Green Master (Rox) PCR system (Roche, Basel, Switzerland) according to the manufacturer’s protocol. Cycle threshold (Ct) values were acquired. The change in mRNA expression was quantified using Ct values compared to the internal control, and the fold change was calculated by 2^-ΔΔCt^. Three independent replicates were performed.

**Cell growth assay**

The cell suspension was added to 96-well plates at 1×10^3^ cells/100 ul/well, and the OD450 was measured by a CCK-8 kit (Dojindo, Kumamoto, Japan) using a microplate reader. The assays were repeated three times.

**Foci formation assay**

Appropriate cells were plated into 6-well plates (1×10^3^ cells/2 ml/well). Two weeks later, the surviving colonies were fixed with 4% paraformaldehyde, stained with crystal violet, and counted. Three independent replicates were performed.

***In vivo* cell growth assay**

All animal experiments were performed according to the guidelines of the Welfare of Experimental Animals at Sun Yat-Sen University Cancer Center. Stable cell lines with overexpression /knockdown (KD) of NRIP3 (2×10^6^) and control cells were subcutaneously injected into the dorsal flanks of nude BALB/c mice (4-week old, male). Animals were grouped randomly (overexpression: n=7; knockdown: n=6). The size of xenografts was measured twice a week. Xenograft volume was calculated by the formula (V = 0.5×L (length) ×W^2^ (width)). Animals were sacrificed 4 weeks later. The xenografts were excised, weighed, fixed, and embedded in paraffin for hematoxylin-eosin (HE) staining and IHC experiments.

**Lipidomics analysis**

A total of 8×10^6^ cells were synchronized by starving for 24 hours and then cultured in DMEM with 10% FBS for 24 hours. Cells were rinsed and extracted with a mixture of methanol and chloroform (1:2). Untargeted lipidomics was performed using a Dionex Ultimate 3000 chromatographic system (Thermo Fished Scientific, San Jose, CA) coupled to a Q-Exactive mass spectrometer (Thermo Fished Scientific, San Jose, CA). Analyses were performed on 5 independent samples for each group. Data were acquired by Xcalibur 4.1 software, and lipid metabolites were identified by LipidSearch 4.0 (Thermo Fished Scientific, San Jose, CA).

**Cycloheximide chase assay**

The cells were treated with 10 µM cycloheximide, and total protein lysates were collected at different time points and subjected to immunoblotting analysis.

**Cytotoxicity assay**

The cells were seeded into 96-well plates and cell viability and IC_50_ (half maximal inhibitory concentration) values were evaluated in cells treated with different concentrations of cisplatin for 72 hours using a CCK-8 kit (Dojindo, Tokyo, Japan). IC_50_ values were calculated using GraphPad Prism 7.0 (GraphPad Software, La Jolla, CA).

For cells with combination treatment: The cells were plated into 96-well plate. After 12 h, cells were exposed to 1 Gy radiation (X-ray irradiator) and treated with different concentrations of chemotherapeutic drugs (cisplatin and carboplatin).

Clonogenic survival was tested by plating cells into 6-well plates at different densities: KYSE30 (0 Gy, 200 cells; 2 Gy, 400 cells; 4 Gy, 1000 cells; 5 Gy, 10000 cells) and EC109 (0 Gy, 200 cells; 1 Gy, 400 cells; 2 Gy, 1000 cells; 4 Gy, 5000 cells). Cells were allowed to attach for 24 hours before being exposed to different doses of radiation (X-ray irradiator, RS2000, Rad Source, USA). After 10-14 days, colonies were fixed, counted and photographed. The survival fraction curves were fitted to a linear quadratic model using GraphPad Prism 7.0 (GraphPad Software, La Jolla, CA, USA).

For cells with combination treatment: 5×10^3^ cells/well were plated into 6-well plate. After 24 h, the cells were exposed to 2Gy radiation (X-ray irradiator) and treated with different concentrations of chemotherapeutic drugs. Cells were then cultured for 11 days and fixed.

**Supplementary Tables:**

**Supplementary Table 1. Eight total unique interactors displayed by BioGRID.**

| **Symbol** | **Name** |
| --- | --- |
| DDI1 | DNA-damage inducible 1 homolog 1 (S. cerevisiae) |
| ELAVL1 | ELAV like RNA binding protein 1 |
| CFTR | cystic fibrosis transmembrane conductance regulator (ATP-binding cassette sub-family C, member 7) |
| CCDC155 | coiled-coil domain containing 155 |
| DDI2 | DNA-damage inducible 1 homolog 2 (S. cerevisiae) |
| PSMG2 | proteasome (prosome, macropain) assembly chaperone 2 |
| PSMG1 | proteasome (prosome, macropain) assembly chaperone 1 |
| TRIM25 | tripartite motif containing 25 |

**Supplementary Table 2: Patients with ESCC receiving radiotherapy/chemotherapy**

| **Treatment** | **Number of patients** |
| --- | --- |
| chemotherapy | 57 |
| radiotherapy | 33 |
| chemotherapy+radiotherapy | 15 |

Note: The main chemotherapy regimen is TP (paclitaxel and cisplatin) or PF (cisplatin and 5-FU).

**Supplementary figure legends:**

**Supplementary figure 1: NRIP3 is upregulated in ESCC tumor tissues.**

(**A**) NRIP3 expression profile across many tumor samples and normal tissues (Each dot represents expression of samples; red, tumor samples; black, normal samples) (downloaded from GEPIA ([http://gepia.cancer-pku.cn/)) (*](http://gepia.cancer-pku.cn/))%20(*), *P*<0.05; TPM: transcripts per million). (**B**) Summary of western blotting results of Figure 1C. The protein level of NRIP3 was normalized to actin. (Nontumor: pool of nontumor tissues; Nontumor was set as 1) (**, *P*<0.01; compared with nontumor tissues pool).

**Supplementary figure 2: The EdU staining results.**

The cells were stained with EdU and detected by FACS. (**A**, **B**) Representative pictures (**A**) and summary (**B**) of EdU positive cells in 30-Vec and 30-NRIP3 cells. (**C, D**) Representative pictures (**C**) and summary (**D**) of EdU positive cells in 109-Ctrl and 109-sh4 cells. (**, *P*<0.01)

**Supplementary figure 3: Knockdown of endogenous NRIP3 decreases ESCC tumor cell growth.**

(**A**) Representative images of xenografts formed by EC9706-sh2 and control cells. (**B**) Tumor growth and tumor weight were compared between EC9706-sh2 and control cells. (**C**) Representative images of xenografts formed by EC9706-sh4 and control cells. (**D**) Tumor growth and tumor weight were compared between EC9706-sh4 and control cells.

**Supplementary figure 4: The RNA-seq results of NRIP3-KD cells and control cells.**

(**A**) The volcano map of differential genes between EC109-sh2 and control cells. (**B**) The gene set enrichment analysis results of EC109-sh2 vs. control cells.

**Supplementary figure 5: The lipid metabolite profiling results of nontarget lipid metabolism method based on chromatographic system coupled to Q-Exactive mass spectrometry.**

The nontarget lipid metabolite profiling analysis was performed in NRIP3-overexpressing cells and NRIP3-KD cells and their corresponding control cells (n=5 per group) using a Dionex Ultimate 3000 chromatographic system coupled to a Q-Exactive mass spectrometry. (**A)** Content comparison of cellular lipid species in NRIP3-overexpressing and -KD cells with their respective control cells (*, *P*<0.05; **, *P*<0.01; n=5 per group). (**B, C, D**) The differential lipid metabolites are demonstrated by heat map allowing visualization of metabolite concentrations using color coding: TGs (**B**), Cers (**C**), CerGs, MGDG and DGMG (**D**). (TG, triglyceride; Cer, ceramides; CerG, simple Glc series; MGDG, monogalactosyldiacylglycerol; DGMG, digalactosylmonoacylglycerol)

**Supplementary figure 6: NRIP3 upregulation confers resistance to cisplatin and carboplatin**

(**A**) Western blotting analysis of NRIP3 in NRIP3-overexpressing cells and control cells (V, vector; N, NRIP3). β-actin was set as loading control. (**B**) NRIP3-overexpressing KYSE150 and KYSE140 cells were treated with cisplatin, and cell viability was determined by cell growth assay. (**C, D**) NRIP3-overexpressing KYSE30, KYSE150 and KYSE140 cells were treated with carboplatin (**C**) or paclitaxel (**D**), and cell viability was determined by cell growth assay (*, *P*<0.05; **, *P*<0.01).

**Supplementary Figure 7: Comet assay results indicate that NRIP3 upregulation confers resistance to cisplatin and ionizing radiation.**

(**A**) Representative images and summary of comet assay results of 30-NRIP3 and 30-Vec cells treated with cisplatin (3 µg/ml) or IR (4 Gy) (V, vector; N, NRIP3). (**B**) Representative images and summary of comet assay results of 109-sh4 and 109-Ctrl cells treated with cisplatin (2 µg/ml) or IR (4 Gy).

**Supplementary Figure 8: Western blotting results of NRIP3-overexpressing or –KD cells treated with ionizing radiation.**

30-NRIP3, Ec109-sh4 and their corresponding control cells were treated with IR (2 Gy) and cells were pelleted 24 h later. Western blotting analysis was performed. GAPDH was set as loading control.

**Supplementary Figure 9: The combination treatment results.**

(**A**) NRIP3-overexpressing cells were treated with platinum salts and IR (1 Gy), cell growth was determined on day 0 and day 3. (CT: combination treatment) (**B, C**) NRIP3-overexpressing cells were treated with platinum salts and IR (2 Gy), foci formation assay was performed: Representative pictures (**B**) and summary (**C**) (**, *P*<0.01). (cisplatin: KYSE30, 2.5 µg/ml; KYSE150, 3 µg/ml; KYSE140, 0.3 µg/ml) (carboplatin: KYSE30, 3.7 µg/ml; KYSE150, 7.4 µg/ml; KYSE140, 0.74 µg/ml).
