# Supplementary figures and images for: NRIP3 upregulation confers resistance to chemoradiotherapy in ESCC via RTF2 removal by accelerating ubiquitination and degradation of RTF2
Source: Oncogenesis. 2020 Aug 24;9(8):75. doi: 10.1038/s41389-020-00260-4 (PMC7445249; doi:10.1038/s41389-020-00260-4)

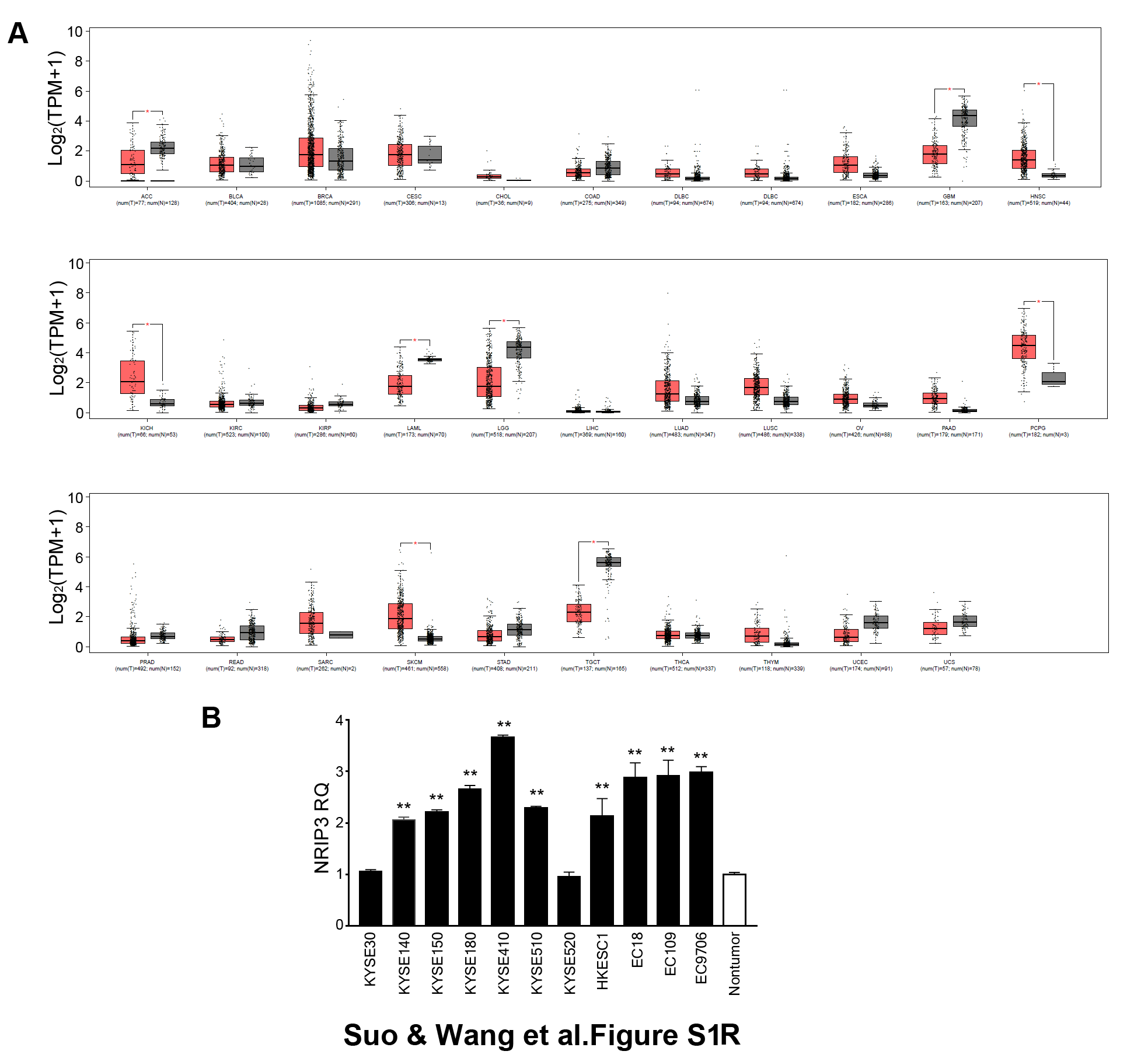

Supplement: Supplementary file 2 — Figure S1 [file 41389_2020_260_MOESM2_ESM.tif]

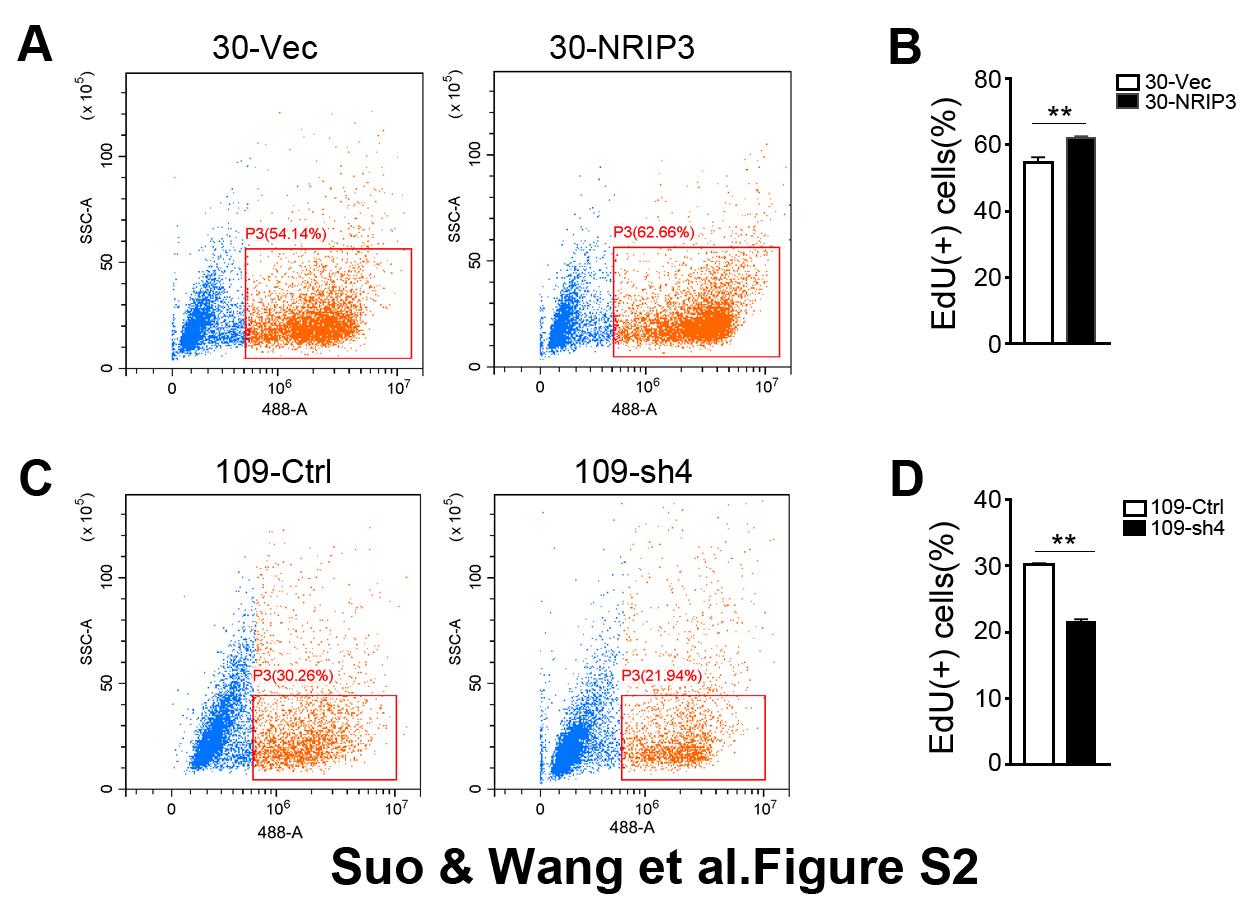

Supplement: Supplementary file 3 — Figure S2 [file 41389_2020_260_MOESM3_ESM.tif]

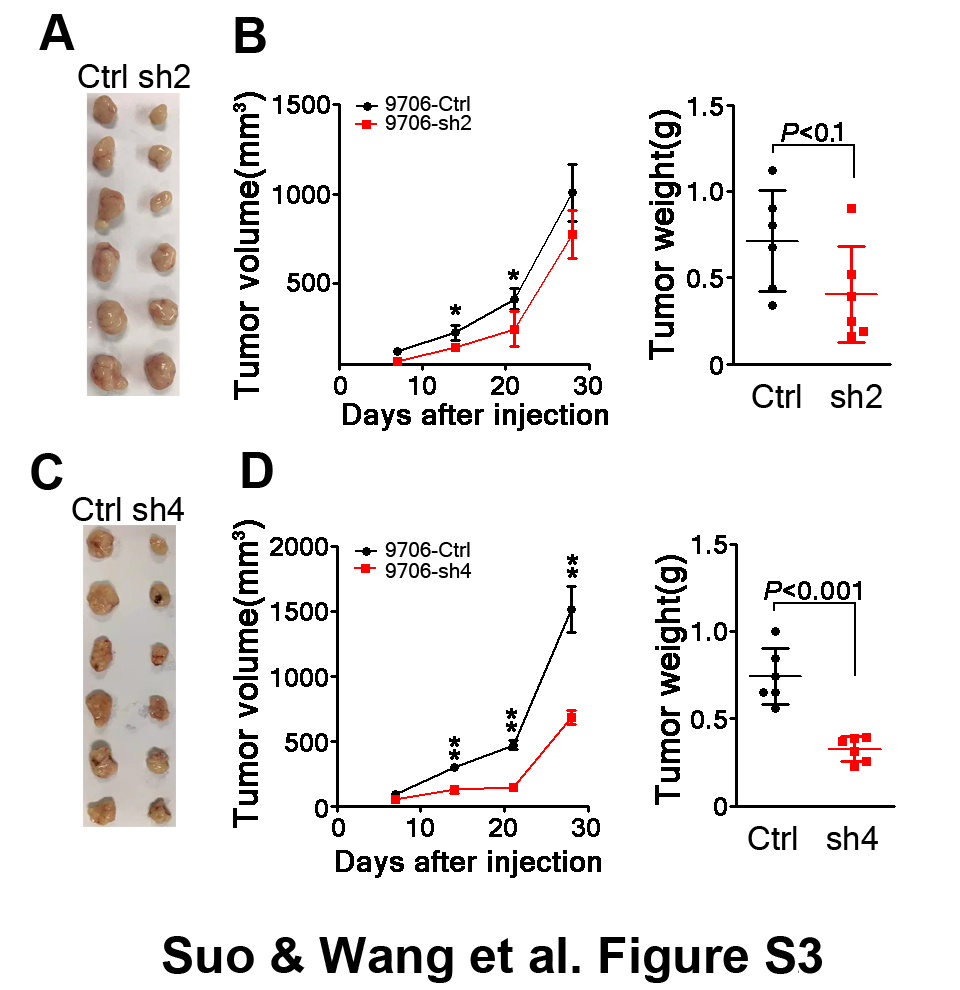

Supplement: Supplementary file 4 — Figure S3 [file 41389_2020_260_MOESM4_ESM.tif]

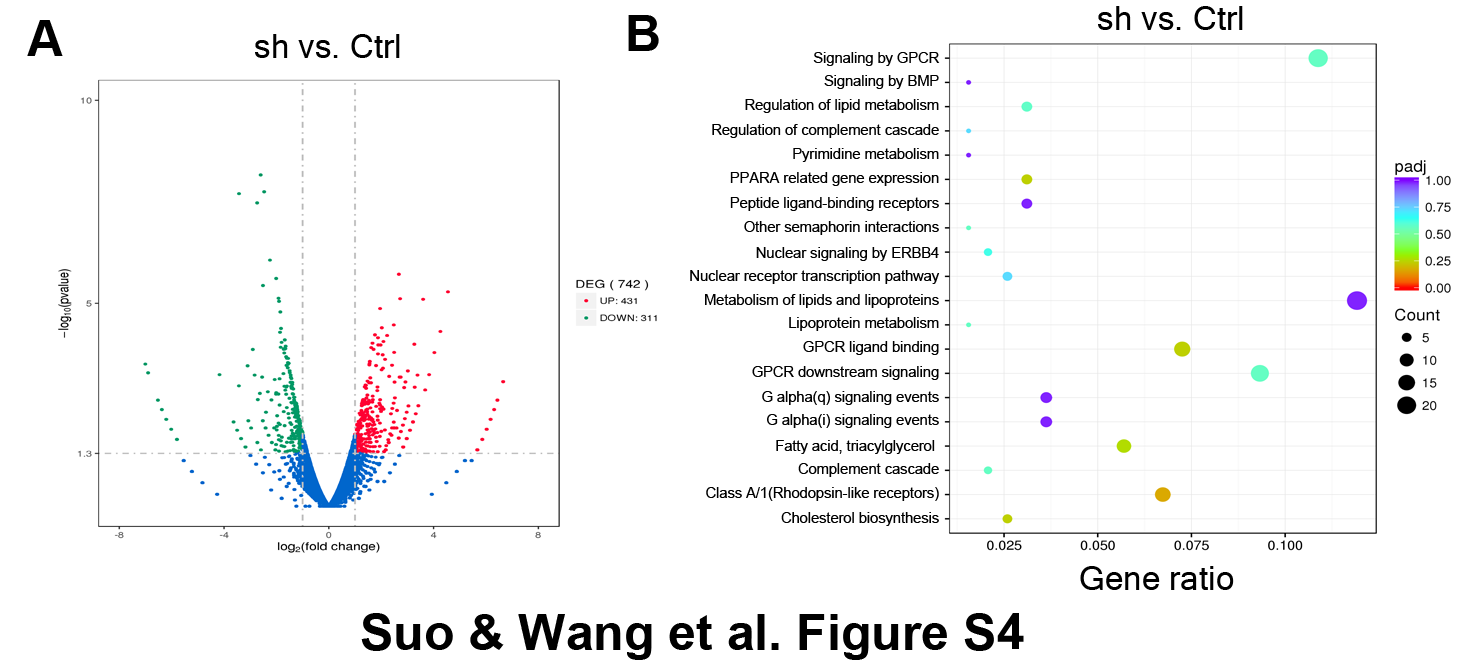

Supplement: Supplementary file 5 — Figure S4 [file 41389_2020_260_MOESM5_ESM.tif]

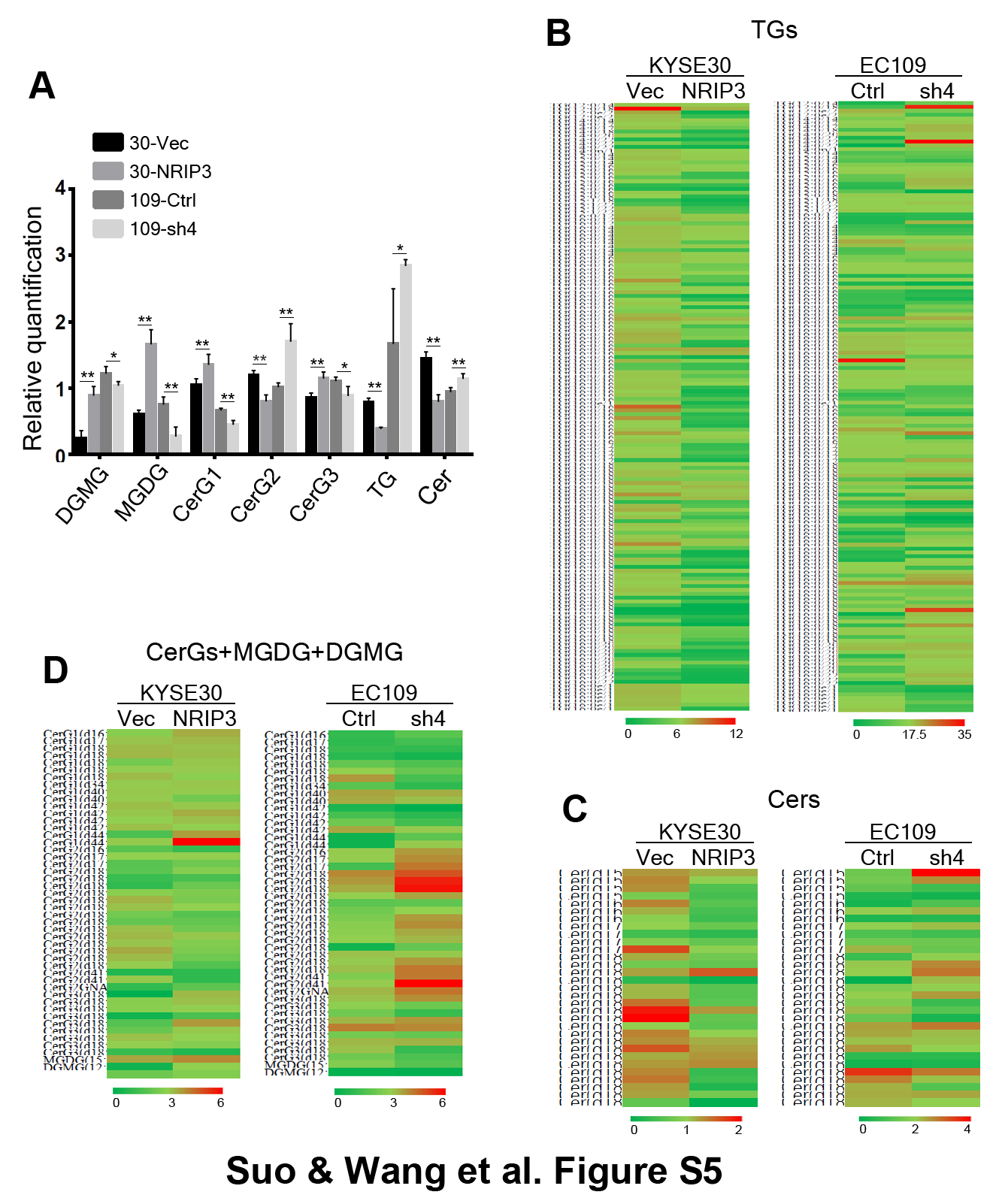

Supplement: Supplementary file 6 — Figure S5 [file 41389_2020_260_MOESM6_ESM.tif]

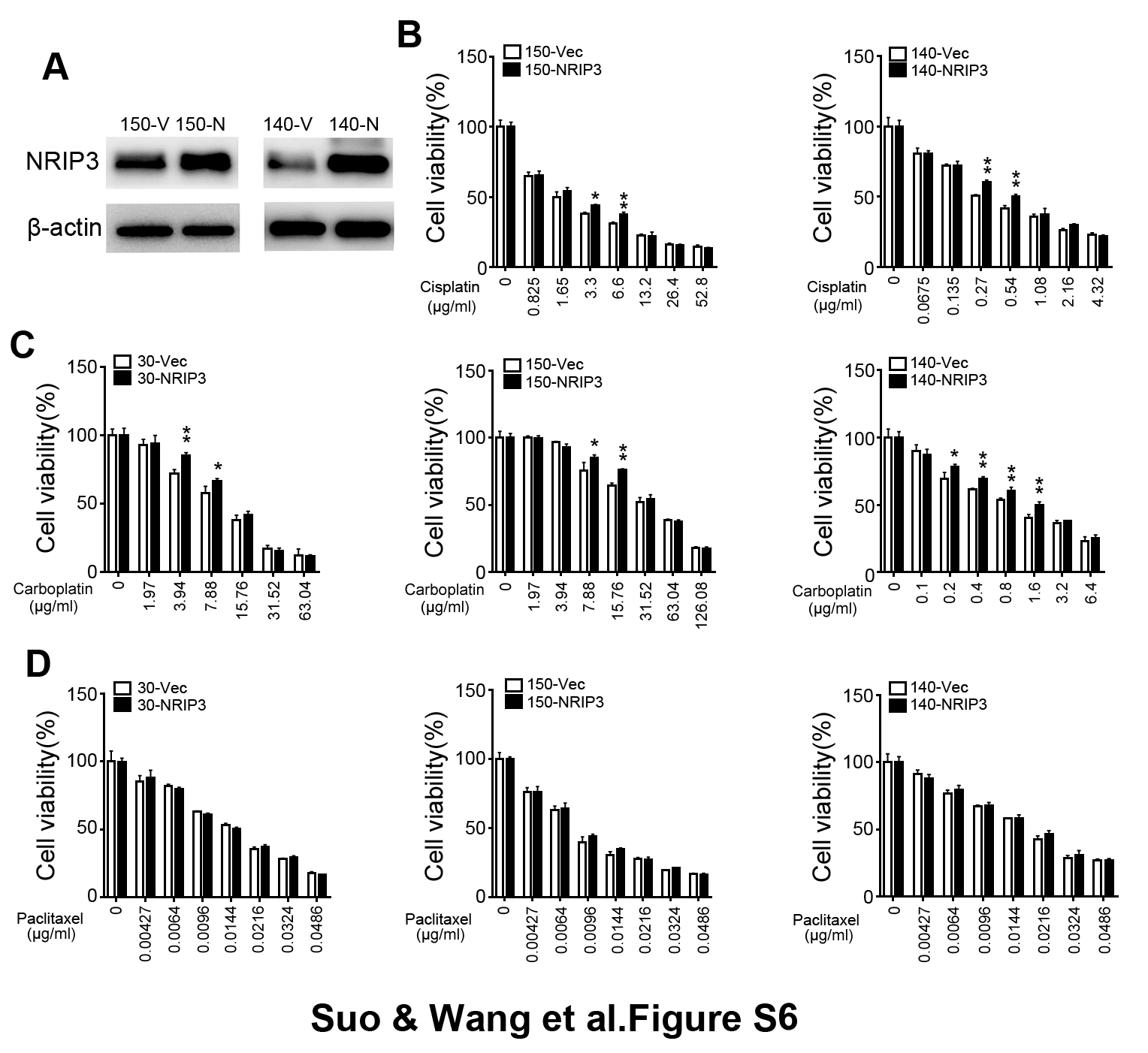

Supplement: Supplementary file 7 — Figure S6 [file 41389_2020_260_MOESM7_ESM.tif]

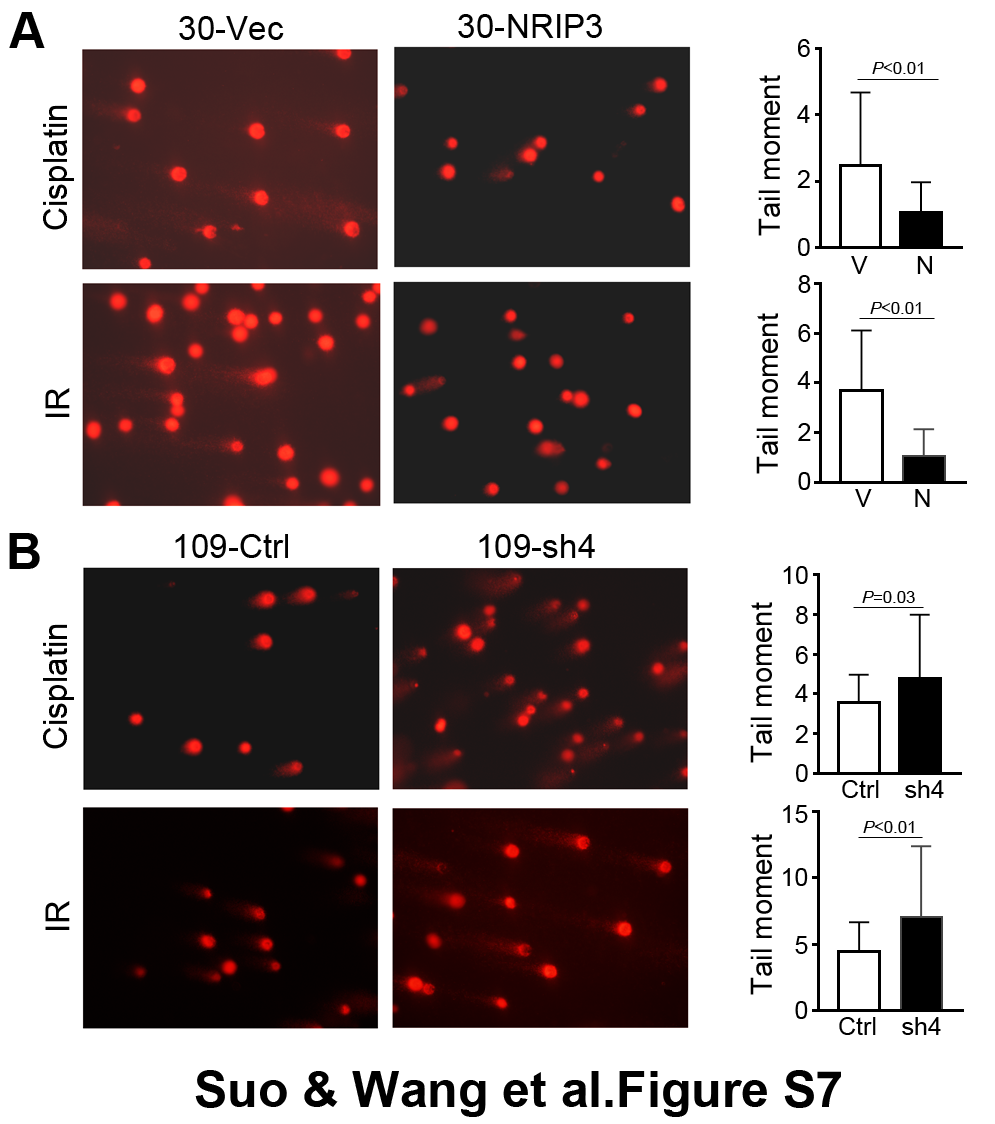

Supplement: Supplementary file 8 — Figure S7 [file 41389_2020_260_MOESM8_ESM.tif]

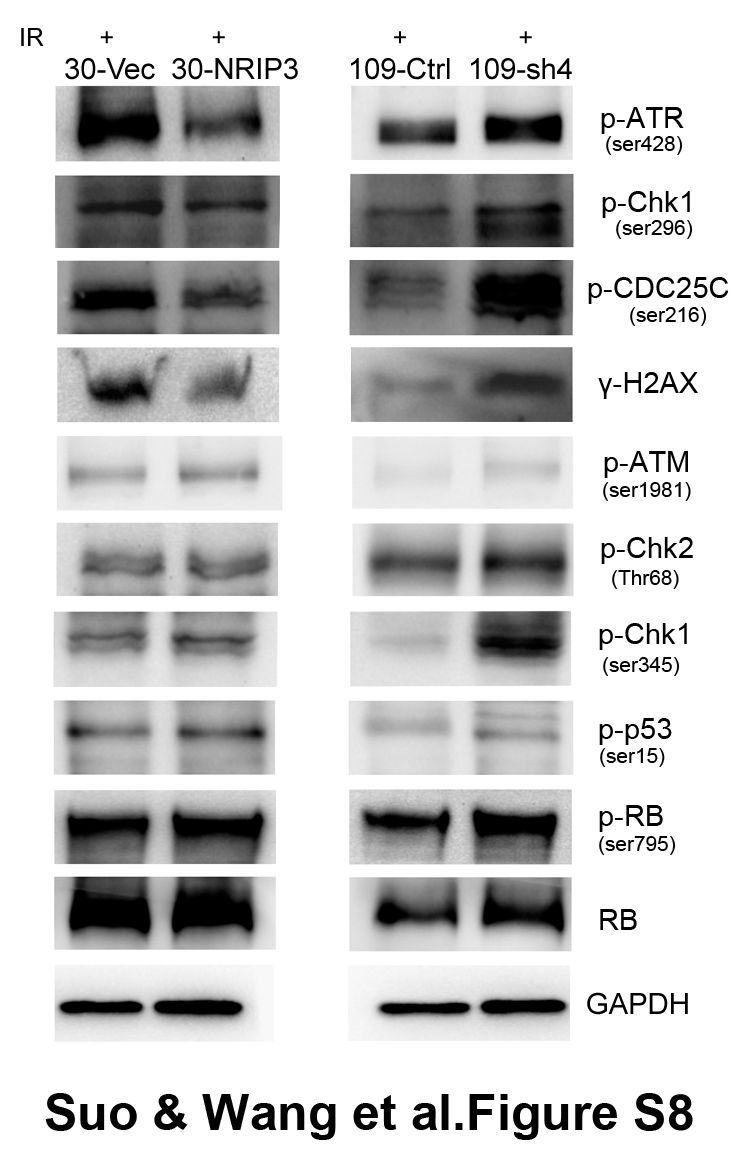

Supplement: Supplementary file 9 — Figure S8 [file 41389_2020_260_MOESM9_ESM.tif]

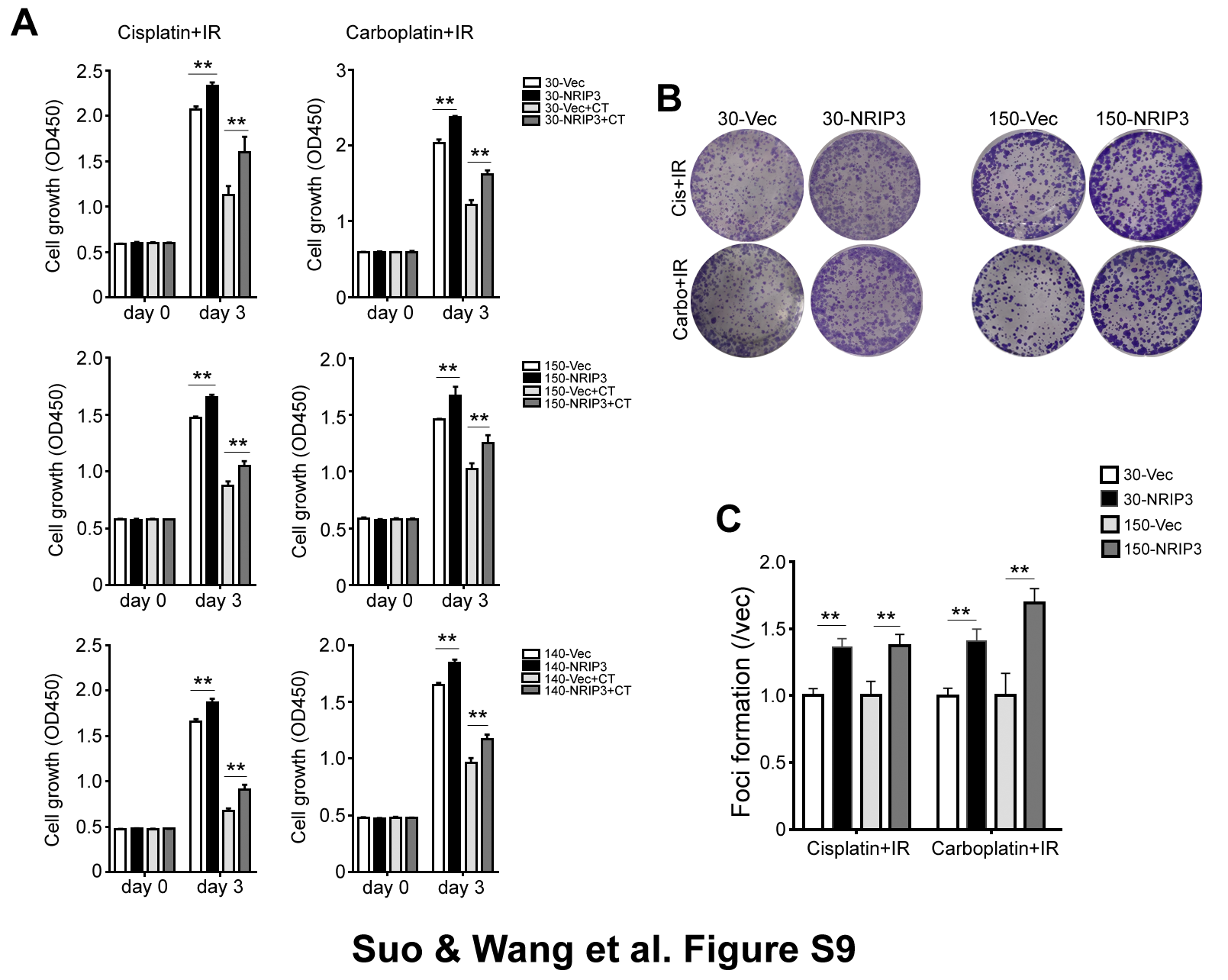

Supplement: Supplementary file 10 — Figure S9 [file 41389_2020_260_MOESM10_ESM.tif]
